# The Effect of a Slowly Rocking Bed on Sleep

**DOI:** 10.1038/s41598-018-19880-3

**Published:** 2018-02-01

**Authors:** Ximena Omlin, Francesco Crivelli, Monika Näf, Lorenz Heinicke, Jelena Skorucak, Alexander Malafeev, Antonio Fernandez Guerrero, Robert Riener, Peter Achermann

**Affiliations:** 1 Sensory-Motor Systems Lab, ETH Zurich, Zurich, Switzerland; 2 Present Address: Sleep and Circadian Neuroscience Institute, Nuffield Department of Clinical Neurosciences, University of Oxford, Oxford, UK; 3 Institute of Pharmacology and Toxicology, University of Zurich, Zurich, Switzerland; 4 Neuroscience Center, University and ETH Zurich, Zurich, Switzerland; 5 Zurich Center for Interdisciplinary Sleep Research, University of Zurich, Zurich, Switzerland; 6 Zurich Center for Integrative Human Physiology, University of Zurich, Zurich, Switzerland; 7 Medical Faculty, University of Zurich, Zurich, Switzerland

## Abstract

Rocking movements appear to affect human sleep. Recent research suggested a facilitated transition from wake to sleep and a boosting of slow oscillations and sleep spindles due to lateral rocking movements during an afternoon nap. This study aimed at investigating the effect of vestibular stimulation on sleep onset, nocturnal sleep and its potential to increase sleep spindles and slow waves, which could influence memory performance. Polysomnography was recorded in 18 males (age: 20–28 years) during three nights: movement until sleep onset (C1), movement for 2 hours (C2), and one baseline (B) without motion. Sleep dependent changes in memory performance were assessed with a word-pair learning task. Although subjects preferred nights with vestibular stimulation, a facilitated sleep onset or a boost in slow oscillations was not observed. N2 sleep and the total number of sleep spindles increased during the 2 h with vestibular stimulation (C2) but not over the entire night. Memory performance increased over night but did not differ between conditions. The lack of an effect might be due to the already high sleep efficiency (96%) and sleep quality of our subjects during baseline. Nocturnal sleep in good sleepers might not benefit from the potential facilitating effects of vestibular stimulation.

## Introduction

Although restorative sleep represents a key factor for well-being and day-time functioning, achieving it often poses a problem. Occasional sleep problems are widely spread and affect about one third of the adult population^1^ and in 10–12% sleep disruption is severe enough to meet diagnostic criteria for clinical insomnia disorder^2^. The importance of sleep is illustrated by the negative effects of chronic sleep loss in humans^3^. Impaired sleep is associated with reduced quality of life^4^ and physical complaints^5^, deteriorates cognitive functioning and has negative impact on emotion regulation^6,7^. Due to the increasing prevalence of sleep complaints^8^ and their impact on health and quality of life, an effective treatment of sleep disturbances is of great relevance. As sleep medication may cause side effects such as dependency or changes in sleep architecture^9,10^, non-pharmacological interventions are of interest. Relaxation techniques, warm feet, and music are popular among the general population to enhance sleep^11,12^. Furthermore, rocking movements appear to have an effect on sleep. For centuries, rocking has been used to promote sleep in babies or toddlers and also sleep in adults seems to be affected by vestibular stimulation^13,14^.

A tendency to shorter sleep latencies and a reduction in the percentage of sleep stage 2 was found for nocturnal sleep in a moving parallel swing bed (lateral movement)^15^. Furthermore, Bayer *et al*. found an accelerated sleep onset as well as a facilitated transition from wake to deeper sleep stages during an afternoon nap in a rocking bed (lateral movement)^14^. The authors also reported an increase in spindle power and slow wave activity indicating that rocking might improve sleep by boosting slow oscillations (SO) and sleep spindles^14^. Sleep in general is assumed to favor memory consolidation (for a review see^16^) and increased sleep spindles and SO especially, seem to be related to an improvement in memory performance^17–21^. However, the mechanisms responsible for these memory benefits are still controversially discussed and not established yet (for a review see^16^).

The aim of this study was, therefore, to investigate the effect of rocking movements on sleep onset and sleep. It was studied whether vestibular stimulation mainly affects sleep onset or if a cumulative effect would be observed with longer stimulation duration. Furthermore, it was of interest to assess if potential beneficial effects are only observable while stimulation is applied or whether a previous period of stimulation has a lasting effect beyond the duration of the stimulation. In addition, it was investigated if vestibular stimulation has the potential to increase sleep spindles and SO, which might influence memory performance assessed by a word pair task. Rocking axis and frequency were selected by each subject prior to the study. In previous studies movement parameters were set by the experimenter and only linear lateral swing movements had been applied^14,15^. It is, therefore, not clear, which kind of movement (translations or rotations, amplitudes, frequencies) could promote relaxation in an optimal way and might facilitate sleep onset and influence sleep quality. A previous study of our lab, investigating the effect of different movement axis on relaxation, showed that movement preferences are subjective^22^. Hence, if the effect of vestibular stimulation is related to relaxation, the greatest effect would be expected with a movement perceived as most relaxing by the participants.

## Methods

### Subjects

Eighteen, healthy, right handed, male subjects (age: 20–28 years; mean: 23.7 years) participated in this study. All subjects were, non-smokers, and free of drugs and medication. Subjects underwent a telephone and questionnaire screening to exclude sleep disorders, diseases of the vestibular system, neurological, psychiatric or acute/chronic internal diseases as well as irregular sleep wake rhythms. Further exclusion criteria were excessive daytime sleepiness measured by the Epworth Sleepiness Scale (cut off: 10)^23^ and susceptibility to motion sickness measured by the short form of the Motion Sickness Susceptibility Questionnaire^24^. Subjects were normal sleepers (habitual sleep duration approximately 8 hours) with moderate alcohol and caffeine consumption (<7 alcoholic drinks/week, <5 beverages or food containing caffeine/day). Sleep quality, sleep efficiency (> 80%) and the absence of sleep disorders were verified during a screening night (polysomnography) prior to the study. The study was approved by the Institutional Review Board of the Swiss Federal Institute of Technology in Zurich (ETH Zurich) and was performed in accordance with the approved guidelines. Written informed consent was obtained from all subjects.

Based on the data (sleep latency, number of spindles) of Bayer *et al*.^14^ (n = 10) we performed a power analysis (G*Power^25^; assumption: correlation between groups = 0.5). It revealed that a sample size of 18 would have 80% power to detect similar effect sizes as observed in the study of Bayer *et al*.^14^.

### Vestibular Stimulation

**Figure 1:**
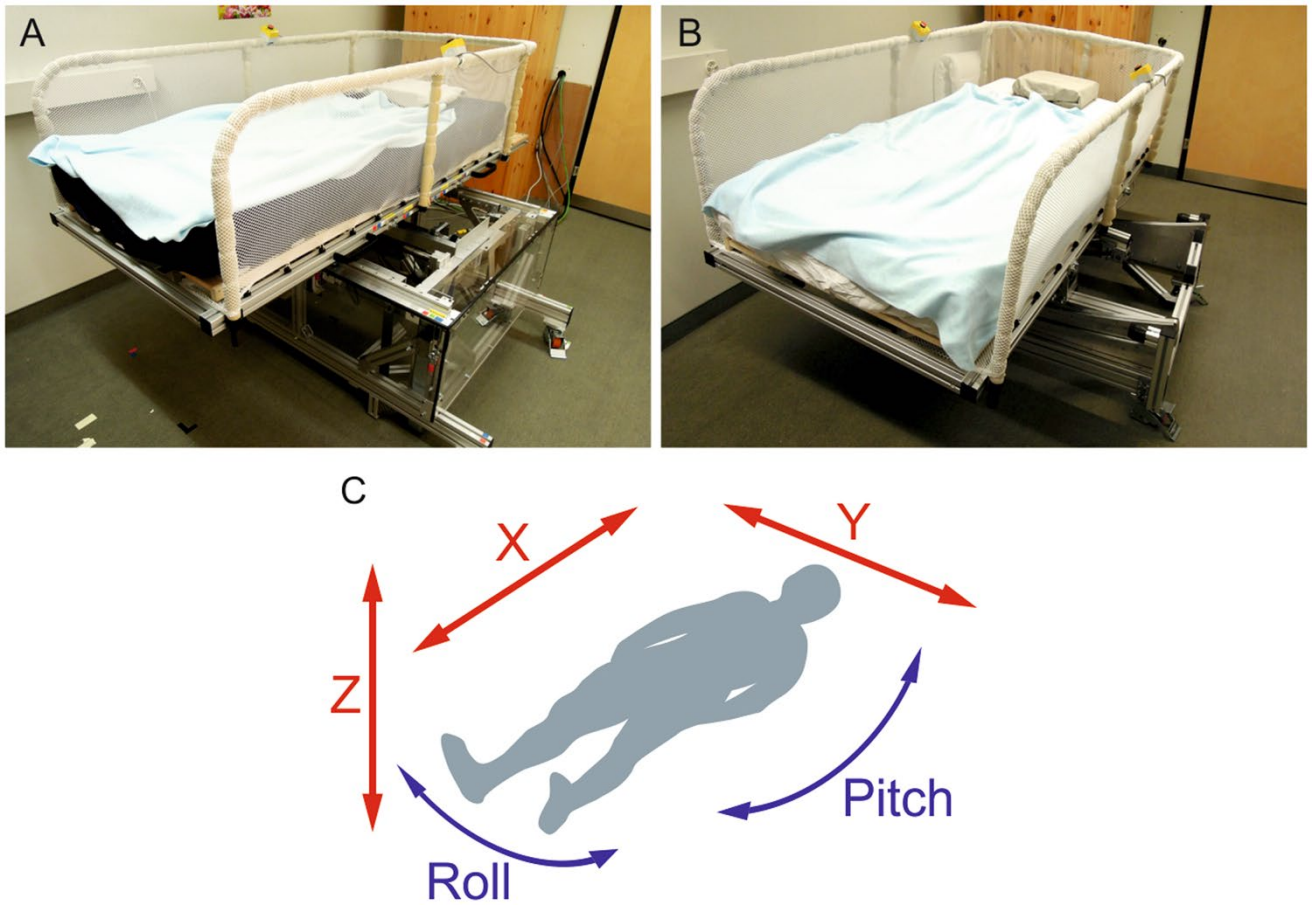
The two actuated platforms and movement axes. (**A**) platform able to provide movements along three translational axes (x-, y-, z-axis). (**B**) platform able to provide movements along two rotational axes (roll-, pitch-axis). (**C**) movement axes: longitudinal (x), lateral (y), and vertical (z) translations (red) and the lateral (roll) and longitudinal (pitch) swing like rotations (blue).

Vestibular stimulation was applied by two actuated bed platforms, composed of a conventional bed, in form of a mattress and a slatted frame, mounted on a moving platform (Fig. 1). Both platforms were actuated by a motor, and controlled by a control program implemented in Matlab/Simulink (Matlab 2013b, MathWorks, Natick, USA). To ensure noise levels below 35 dB, the electrical cabinet and control PC were placed outside the bedroom. Platform A was able to perform three linear translations along the longitudinal (x), lateral (y), and vertical (z) body axes. Platform B allowed two swing-like rotations along longitudinal (pitch) and lateral (roll) direction (Fig. 1). To avoid or minimize artefacts induced by the movement of the platform, the measurement equipment was placed on the platform and the cables were guided along the platform. The occurrences of electromagnetic interferences as well as movement induced artefacts were tested and could be excluded as no perturbation were observed for movements along the z-, roll-, pitch-axis and only small (< 1 µV) ones were found for those along the x-, and y-axis.

### Movement Selection

Subjects could select their preferred movement during the screening night. Subjects could choose between five movement axes (x-, y-, z-, roll-, and pitch-axis). Furthermore, subjects selected between two frequencies a faster (0.24 Hz) and a slower one (0.16 Hz). To ensure comparable maximal velocities (0.1 m/s) for both frequencies, the amplitudes were adapted accordingly. Amplitude for the fast frequency was 0.066 m and for the slow frequency 0.10 m. Subjects tested each movement for 2 minutes while lying in bed in a darkened room with closed eyes. Movement axes and frequencies were applied in randomized order. After each movement subjects gave feedback and ranked the movements during a short break.

### Experimental Protocol

Polysomnography was recorded in each subject during three experimental nights in the sleep laboratory (at weekly intervals): one baseline night (B) without rocking and two nights with rocking (C1 and C2). Subjects had to adhere to regular bed times for 7 days prior to the experimental night and throughout the entire experimental phase (three weeks in total). Bed times were selected by the subjects according to their habituation (between 10–12 pm) and stayed the same for all experimental nights. Time in bed was eight hours (subjects’ habitual sleep duration) and subjects were allowed to shift their bedtimes at maximum by 30 minutes during the nights not spent in the laboratory. Furthermore, subjects had to abstain from caffeine and alcohol during the 3 days prior to each experimental night. Compliance with the bedtimes was assessed by actigraphy and sleep logs. The actimeter was worn on the left wrist and measured arm movements allowing to establish subjects’ rest-activity rhythm. Abstention from alcohol consumption was verified by a breath alcohol test.

Vestibular stimulation was started with lights out. For condition C1 vestibular stimulation was applied until sleep onset in order to investigate whether vestibular stimulation is only effective during the transition from wake to sleep. Vestibular stimulation was switched off after the first occurrence of 3 consecutive epochs of stage 2 sleep (N2). Sleep stages were visually determined according to standard criteria^26^. For condition C2 vestibular stimulation was applied for the first 2 hours in order to investigate if with prolonged stimulation a cumulative effect is present. Furthermore, it was of interest whether a previous period of prolonged stimulation has a lasting effect beyond the duration of stimulation. The duration of the C2 conditions (2 hours) was selected to cover approximately the first sleep cycle and therefore, expose the participant to the stimulation across different sleep stages. Vestibular stimulation was switch off 2 hours after lights out, independent of the sleep stage the subject was in. The order of the experimental conditions was balanced and conditions were randomly assigned to the participants. To ensure the same noise level for all conditions, pre-recorded platform noise was presented during the first 2 hours after lights out in condition B and from sleep onset until 2 hours after lights out in condition C1. Pre-recorded platform noise matched the selected movement axis as well as the chosen movement frequency. Noise levels during stimulation and during pre-recorded noise presentation were below 35 dB. Subjects were informed five minutes prior to lights out whether vestibular stimulation was applied or not. However, in case of a stimulation night they were not informed about the exact duration of stimulation but informed that the stimulation would stop at a random point in the course of the night. This protocol was used to minimize subjects’ potential bias (expectations) towards the experimental conditions, which could have had an influence on the process of falling asleep as well as on the subjective rating of sleep quality. After the last experimental night subjects had to compare stimulation against no stimulation and state which condition they preferred.

### Declarative Memory Task

A word-pair learning task was used to assess declarative memory performance. Word-pair tasks are suitable to determine declarative memory performance in the context of sleep, as they are sensitive to effects of sleep^27,28^. Prior to sleep, subjects performed a word-pair memory task consisting of 40 semantically related word pairs, which were presented in randomized order. Three different word pair lists were used for the three nights. The nouns of the different word pair lists were standardized and matched in terms of length, concreteness, emotionality, and meaningfulness^28^. The list versions were randomized and balanced over the experimental conditions and nights.

Subjects had to learn the word pairs, were tested immediately after learning (immediate recall) and after 8 hours of sleep in the morning (delayed recall). Word pairs were presented on a computer screen for 4 s each. During recall subjects had to recall the second word after the first word of the pair was shown. There was no time limit to answer, but subjects were instructed to respond as fast as possible. After subjects entered the second word, the correct word pair was shown again for 2 s as a feedback. The times of the word pair learning and testing were standardized (one hour before bed time, 30 min after waking up) in order to keep the time between acquisition and sleep short and to reduce the effect of sleep inertia on cognition in the morning^29–31^.

Each correct word pair was scored with one point and correctly recalled word pairs containing mistakes (plural/singular form, spelling) with half a point. Overnight performance improvement was defined as the difference in correctly recalled word pairs between immediate and delayed recall. Initial acquisition rate, indicating how much of the individual learning capacity is already achieved in the immediate recall, was calculated as the performance in the immediate recall expressed as the percentage of the delayed recall performance^32^.

### Physiological Recordings & Data Analysis

EEG (according to the 10–20 system: F3, F4, C3, C4, P3, P4, O1, O2, A1, A2, referenced to Cz), submental EMG and EOG were continuously recorded throughout the entire 8 h sleep period with a polygraphic amplifier Artisan (Micromed, Mogliano, Veneto,Italy). The signals were sampled at 256 Hz and recorded with the software Rembrandt DataLab (Version 8.0; Embla Systems, Broomfield, CO, USA). Analogue signals were filtered with a high pass filter (EEG: −3 dB at 0.15 Hz; EMG: 10 Hz; ECG: 1 Hz) and an anti-aliasing low-pass filter (−3 dB at 67.2 Hz). For further analysis, the EEG signals were re-referenced to the contra-lateral mastoid (A1, A2). The sleep stages were scored visually on a 20-s epoch basis according to standard criteria^26^. For artefact removal, artefacts were identified visually and with semi-automatic artefact detection (see^33^ for details).

EEG power in specific frequency bands (derivation C3-A2; delta: 0.75–4.5 Hz; theta: 4.5–9 Hz; alpha: 9–15 Hz; sigma: 11–15 Hz; beta: 15–25 Hz) was calculated based on spectral analysis performed with an FFT (Hanning window; averages over 5 4-s epochs) and matched with the sleep stages. Spindle detection (derivation C3-A2) was performed using a spindle detection algorithm according to Ferrarelli *et al*.^34,35^ applied to all artefact-free NREM sleep epochs. EEG signals were band-pass filtered between 12 and 15 Hz (−3 dB at 12 and 15 Hz), rectified and the thresholds for spindle detection and spindle duration were estimated (lower threshold = 2 * average amplitude; upper threshold = 6 * average amplitude). A spindle was detected whenever the signal amplitude exceeded the upper threshold (six times the average amplitude).

Detection of slow waves (derivation F3-A2) was performed using an algorithm previously described^36^. EEG data (0.5–2 Hz) were down sampled to 128 Hz and band pass filtered (third-order Chebyshev type II high-pass filter; −3 dB at 0.4 Hz; sixth-order Chebyshev type II low-pass filter, −3 dB at 2.3 Hz). Phase distortion was prevented by filtering in forward and reverse direction. Slow waves were subdivided in positive and negative half-waves with an amplitude of ≥37.5 µV (75 µV peak to peak amplitude^26^) and were defined as positive or negative deflections between consecutive zero-line crossings.

To assess whether rocking had an impact on sleep continuity and sleep quality the number of excluded epochs (artefacts) and sleep stage changes were quantified from sleep onset until 2 hours after lights out.

### Statistical Analysis

#### EEG Features

All EEG features were statistically analysed using a univariate general linear model followed by post hoc least significant difference tests (LSD-tests). EEG measures (sleep latency, sleep stages, total power in frequency bands, number of SO and spindles, SO and spindle density, excluded epochs, sleep stage changes) entered the model as dependent variable, condition as fixed factor and subjects as random factor. The conditions with movements were compared among each other and to baseline. EEG data were analysed for the entire night and the first 2 hours after lights out. The significance level was set at p < 0.05. The statistical analysis was performed with the SPSS software (SPSS Inc., Chicago, Illinois, USA).

#### Word-Pair Task

Data of 16 subjects were included in the statistical analysis of the word-pair task. Two subjects recalled all 40 word pairs reaching the maximum points and were therefore excluded due to a ceiling effect. Statistical analysis was performed using a linear mixed model with random effects. Word-pair task performance measures (overnight memory improvement, immediate recall, delayed recall, initial acquisition rate) entered the model as dependent variable, the condition as fixed effect and subjects as random effects. To exclude effects of the word-pair task list version or the experimental night, interactions between these factors and the conditions were tested. To select the model with optimal fit an F-test with a Kenward-Roger correction was applied. Linear mixed model analysis was performed with the lme4 package^37^ using the statistical software R^38^. Post-hoc comparisons were performed by a Tukey’s test using the glht-method (general linear hypothesis testing) of the multcomp package^39^. Associations between sleep spindles, slow oscillation measures and word-pair task performance measures were determined using Pearson’s correlation coefficients (two-tailed). EEG measures of the entire night as well as only the first two hours of the recording were included in the analysis.

## Results

### Movement Selection

**Figure 2:**
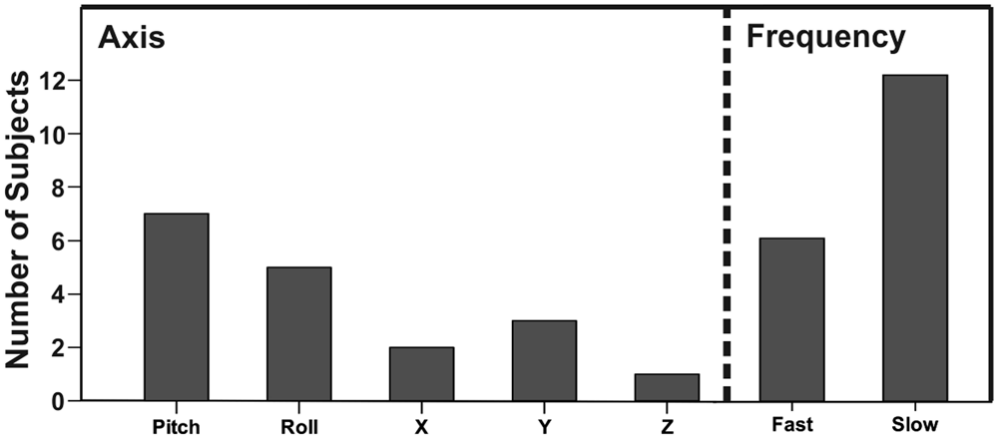
Movement selection. Left: preferred movement axis. Right: preferred movement frequency (fast: 0.24 Hz; slow: 0.16 Hz).

A preference for rotational movements was observed as 12 out of 18 subjects selected either a pitch-, or roll-axis. Furthermore, the majority of the subjects (n = 12) decided upon a slow stimulation frequency of 0.16 Hz rather than the faster frequency of 0.24 Hz (Fig. 2). Most subjects preferred the conditions with movement (12 preferred motion night, 2 preferred baseline night, 4 had no preference; assessed after the last experimental night).

### Sleep Architecture

Sleep latency (lights out to first occurrence of N2), slow wave sleep (SWS) latency and REM sleep latency were comparable among the conditions and did not reveal significant differences. Sleep efficiency, N1, N3 and time awake after sleep onset (WASO) did not differ between the conditions when comparing the data over the entire night (8 hours) as well as during the first 2 hours after lights out. N2 sleep (mean (SD), p-value) was significantly increased in C2 (52.41 (11.45) min) compared to B (46.09 (13.00) min, p = 0.005) and C1 (46.56 (9.16) min, p = 0.008) during the first 2 hours after lights out, but not for the entire night (Table 1). In addition, when considering the first 3 hours after lights out the differences in N2 sleep were no longer present (B: 79.61 (20.19) min; C1: 75.61 (17.55) min; C2: 77.11 (17.41) min).

**Table 1:** Mean and standard deviation of sleep variables derived from visual scoring. Values were calculated over the entire night (normal font) and over the first 2 hours after lights out (in italics).

	B	C1	C2
Sleep latency [min]	8.48 (5.80)	9.89 (5.68)	9.67 (5.43)
SWS latency [min]	11.09 (1.33)	12.11 (1.25)	10.35 (0.84)
REM sleep latency [min]	66.63 (12.01)	68.80 (15.83)	75.33 (24.53)
Sleep efficiency [%]	96.77 (4.22)	96.50 (3.07)	96.75 (2.39)
	*91.73 (7.43)*	*90.82 (6.18)*	*91.39 (4.66)*
N1 [min]	10.54 (7.79)	13.87 (12.89)	12.89 (11.57)
	*1.50 (1.52)*	*2.00 (2.04)*	*2.31 (3.00)*
N2 [min]	232.50 (37.11)	232.89 (33.34)	238.67 (28.63)
	*46.09 (13.00)*	*46.56 (9.16)*	52.41 (11.45)* ^*a,b*^
N3 [min]	88.24 (23.92)	89.15 (25.58)	85.41 (22.36)
	*50.98 (12.11)*	*48.93 (10.79)*	*46.20 (10.19)*
WASO [min]	6.87 (16.52)	5.33 (8.48)	5.81 (9.40)
	*1.44 (4.48)*	*1.13 (3.73)*	*0.67 (1.20)*

Experimental conditions: Baseline (B), vestibular stimulation until sleep onset (C1), vestibular stimulation for 2 hours after lights out (C2); sleep latency: lights out to first occurrence of N2; SWS latency: sleep onset to first occurrence of N3; REM sleep latency: sleep onset to first occurrence of REM sleep; WASO = wake after sleep onset. *= p ≤ 0.05 (LSD-test), a: difference between C2 & C1, b: difference between C2 & B.

Conditions did not differ significantly in the number of excluded epochs (B: mean: 40.11 (standard deviation: 21.32); C1: 36.28 (16.38); C2: 31.90 (13.50)) or the number of sleep stage changes (B: 24.39 (12.65); C1: 26.17 (18.98); C2: 28.83 (23.48)).

### Spectral Analysis

**Figure 3:**
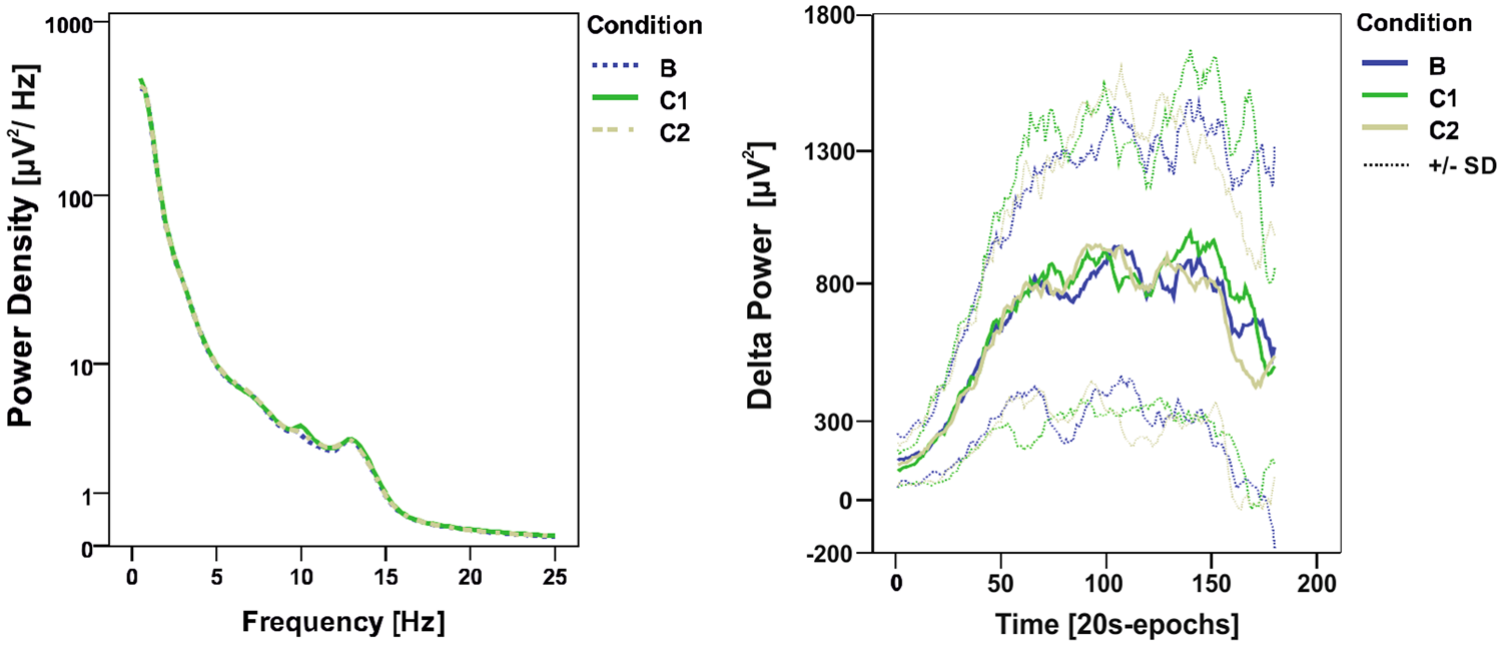
Average power density spectra and build-up of delta power. Left: power density spectra averaged over NREM sleep of the entire night in all three experimental conditions. Right: build-up of delta power and standard deviation (SD) during the first hour after sleep onset (20-s epochs, moving average over 7 epochs). (Experimental conditions: Baseline (**B**), vestibular stimulation until sleep onset (C1), vestibular stimulation for 2 hours after lights out).

Spectral power did not differ between the experimental conditions (Fig. 3 left; Table 2). Delta, theta, alpha, sigma, and beta power of the entire night and the first 2 hours after lights out revealed no differences between conditions (Table 2).

**Table 2:** Mean and standard deviation of spectral power in different frequency bands. Values were calculated over the entire night (normal font) and over the first 2 hours after lights out (in italics).

	B	C1	C2
Delta Power [µV^2^]	366.13 (119.77)	370.87 (128.22)	375.47 (137.34)
	*569.72 (214.98)*	*579.39 (212.66)*	*543.58 (196.22)*
Theta Power [µV^2^]	22.58 (7.20)	23.12 (8.61)	23.47 (9.25)
	*29.11 (10.16)*	*29.99 (12.36)*	*29.769 (11.95)*
Alpha Power [µV^2^]	20.73 (7.81)	21.95 (11.88)	21.27 (8.34)
	*24.28 (15.30)*	*26.23 (22.14)*	*24.43 (12.11)*
Sigma Power [µV^2^]	7.22 (2.35)	7.50 (2.78)	7.26 (2.10)
	*6.86 (2.32)*	*7.21 (2.82)*	*7.08 (2.29)*
Beta Power [µV^2^]	3.02 (1.66)	3.14 (1.69)	3.04 (1.51)
	*2.94 (1.44)*	*3.20 (1.52)*	*3.25 (1.55)*

Experimental conditions: Baseline (B), vestibular stimulation until sleep onset (C1), vestibular stimulation for 2 hours after lights out (C2). Delta band: 0.75–4.5 Hz; Theta band: 4.5–9 Hz; Alpha band: 9–15 Hz; Sigma band: 11–15 Hz; Beta band: 15–25 Hz. No significant differences were observed.

Delta power was further subdivided in three frequency ranges: oscillations <1 Hz, between 1–2 Hz and between 2–4 Hz. However, spectral power in these frequency bands did not differ between conditions neither when considering the entire night, nor when considering only the first 2 hours after lights out. Furthermore, the temporal evolution of delta power (build up) during the first hour after sleep onset was similar in all conditions (Fig. 3, right).

### Sleep Spindles and Slow Oscillations

**Figure 4:**
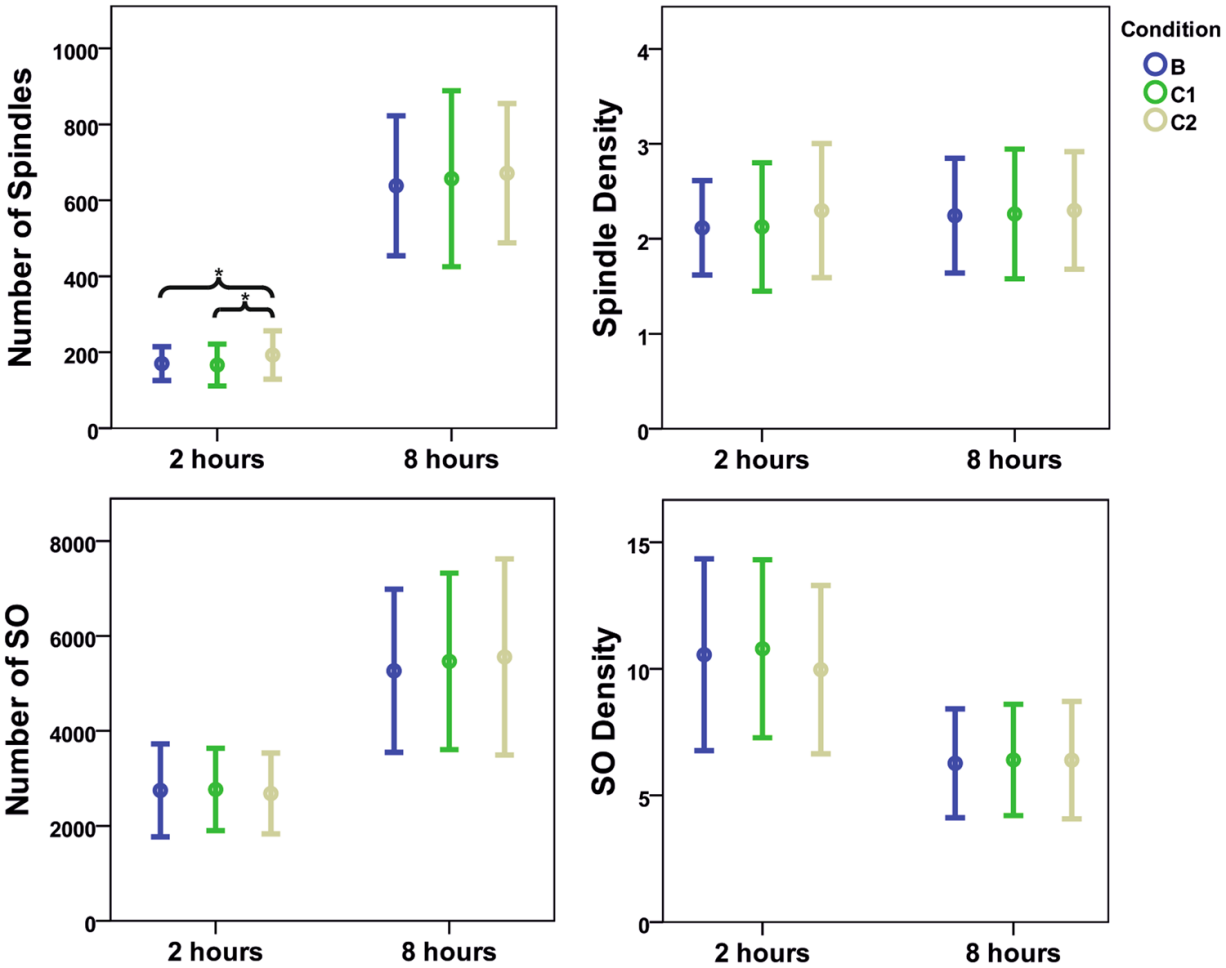
Number and density (mean and standard deviation) of spindles and slow oscillations (SO) of all conditions. Upper left: number of spindles of the first 2 hours after lights out and of the entire night. Upper right: spindle density (number of spindles per 20-s epoch). Lower left: number of SO of the first 2 hours after lights out and of the entire night. Lower right: density of SO (number of SO per 20-s epoch). Shown are mean values (dots) ± standard deviation (whiskers). (Experimental conditions: Baseline (**B**), vestibular stimulation until sleep onset (C1), vestibular stimulation for 2 hours after lights out); *p ≤ 0.05 (LSD-test).

Total number of sleep spindles (mean (SD), p-value) was significantly increased during the 2 h of rocking (C2; 195.61 (66.56)) compared to B (170.00 (44.39), 0.047) and C1 (166.28 (55.03), 0.024). However, this increase was only present during the first 2 hours after lights out. The total number of sleep spindles did not differ between conditions when considering the first 3 hours after lights out or the entire night (B: 638.22 (184.36); C1: 657.06 (231.84); C2: 671.33 (183.39)). However, spindle density (number of spindles per 20-s epoch) was not influenced by the different experimental conditions neither during the entire night (B: 2.24 (0.61); C1: 2.26 (0.68); C2: 2.30 (0.62)), nor during the first 2 hours (B: 2.12 (0.50); C1: 2.13 (0.68); C2: 2.30 (0.71)). The number of slow oscillations did not differ between the experimental conditions for the first 2 hours (B: 2747.33 (979.71); C1: 2765.94 (868.89); C2: 2681.17 (851.04)) as well as for the entire night (B: 5265.83 (1717.31); C1: 5465.33 (1858.04); C2: 5557.44 (2064.26)). The same was observed for the density of slow oscillation (2 hours: B: 10.56 (3.78); C1: 10.79 (3.51); C2: 9.97 (3.32); entire night: B: 6.27 (2.15); C1: 6.40 (2.20); C2: 6.40 (2.32)) (Fig. 4).

### Word-Pair Task

**Figure 5:**
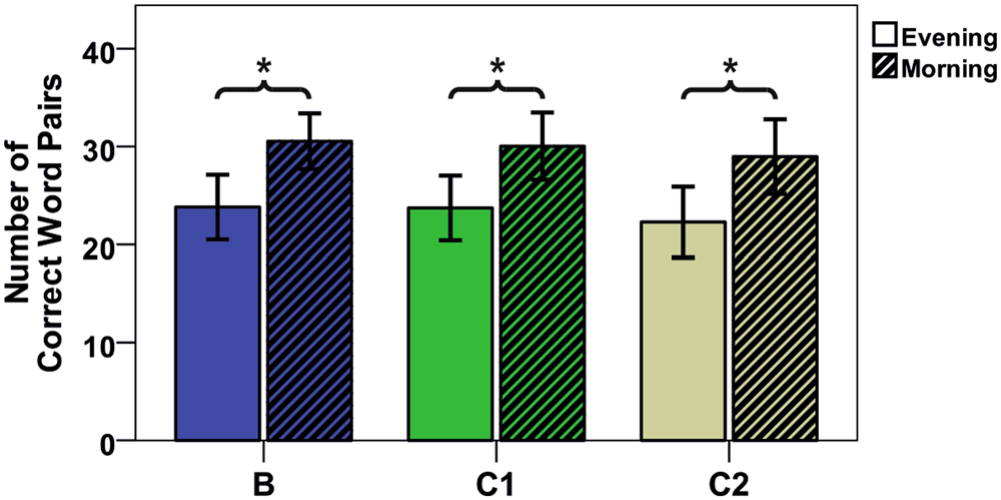
Overnight memory improvement of all conditions. Mean and standard deviation of the number of correctly recalled word pairs in immediate (evening) and delayed (morning) recall (n = 16). (Experimental conditions: Baseline (**B**), vestibular stimulation until sleep onset (C1), vestibular stimulation for 2 hours after lights out); *p ≤ 0.05 (Tukey’s test).

The number of correct recalled word pairs was significantly higher for the delayed recall compared to the immediate recall (Overnight memory improvement (mean (standard deviation), z-value, p-value) = C1: 6.31 (2.75), 9.18, < 0.001; C2: 6.69 (3.43), 7.79, < 0.001); B: 6.72 (3.76), 7.14, < 0.001) (Fig. 5).

However, conditions did not differ. Furthermore, no differences between the experimental conditions were found for overnight memory improvement, immediate recall, delayed recall, or initial acquisition rate.

Spindle power, delta power, number of SO as well as density of slow oscillation were not correlated with overnight performance improvement, immediate recall, delayed recall or initial acquisition rate in any of the experimental conditions. However, spindle number and density correlated significantly with immediate and delayed recall (Table 3).

**Table 3:** Pearson’s correlation coefficients (r-value (p-value)) between spindle power, number and density and word-pair task measures. Values were calculated over the entire night (normal font) and over the first 2 hours after lights out (in italics).

**Condition**	**Overnight Performance Improvement**	**Immediate Recall**	**Delayed Recall**	**Initial Acquisition Rate**
	Spindle Power			
B	0.09 (0.733)	−0.09 (0.748)	−0.04 (0.883)	−0.16 (0.562)
	*0.16 (0.558)*	−*0.26 (0.326)*	−*0.20 (0.458)*	−*0.26 (0.339)*
C1	0.15 (0.573)	−0.14 (0.611)	−0.07 (0.793)	−0.33 (0.219)
	*0.12 (0.648)*	−*0.03 (0.916)*	*0.02 (0.936)*	−*0.25 (0.354)*
C2	0.04 (0.884)	0.05 (0.857)	0.07 (0.812)	−0.05 (0.854)
	*0.06 (0.836)*	*0.10 (0.705)*	*0.12 (0.650)*	−*0.05 (0.847)*
	Spindle Number			
B	−0.43 (0.094)	0.67* (0.005)	0.49 (0.054)	0.58 (0.018)
	−*0.42 (0.106)*	0.66* (0.005)	*0.49 (0.054)*	*0.60 (0.013)*
C1	0.07 (0.799)	0.60 (0.013)	0.61* (0.012)	0.16 (0.562)
	*0.18 (0.495)*	0.63* (0.010)	0.68* (0.004)	*0.09 (0.733)*
C2	0.06 (0.825)	0.65* (0.007)	0.64* (0.005)	0.32 (0.227)
	−*0.14 (0.601)*	*0.60 (0.014)*	*0.505 (0.046)*	*0.38 (0.148)*
	Spindle Density			
B	−0.40 (0.122)	0.64* (0.007)	0.48 (0.059)	0.54 (0.030)
	−*0.32 (0.235)*	*0.49 (0.054)*	*0.36 (0.169)*	*0.46 (0.073)*
C1	0.16 (0.549)	0.59 (0.015)	0.64* (0.008)	0.10 (0.724)
	*0.17 (0.524)*	0.70* (0.003)	0.74* (0.001)	*0.14 (0.607)*
C2	0.13 (0.631)	0.73* (0.001)	0.75* (0.001)	0.29 (0.271)
	−*0.05 (0.859)*	0.63* (0.009)	*0.57 (0.020)*	*0.33 (0.211)*

N = 16; *p ≤ 0.0125 (Bonferroni corrected); two-tailed. Experimental conditions: Baseline (B), vestibular stimulation until sleep onset (C1), vestibular stimulation for 2 hours after lights out. Spindle power: NREM sleep EEG power in 11–15 Hz range; Spindle density: Number of spindles per 20s epoch.

## Discussion

### Sleep

In contrast to the findings of Bayer *et al*.^14^ our applied vestibular stimulation did not shorten sleep onset nor did it facilitate the transition to deep sleep. Sleep latencies (time from lights out until the first appearance of N2 sleep) and SWS latencies (time from sleep onset until first appearance of N3 sleep) did not exhibit any changes due to vestibular stimulation. Also when calculating the N2 latency as the difference between first occurrence of N2 and first occurrence of N1, which was shortened in the study of Bayer *et al*.^14^, no differences between the conditions were observed. Furthermore, spectral analysis did not reveal any differences between the conditions. Spectral power was comparable in all frequency bands including SWA. In contrast to the study of Bayer *et al*.^14^ a boost in SWA was not observed. The build-up of SWA in the first hour after sleep onset followed the same time course in all conditions. Therefore, SWA was neither enhanced nor its build up facilitated by vestibular stimulation. Similarly, the number and density of SO remained stable across the different conditions. We found our effect sizes to be small (Cohen’s d for baseline compared to C1 and C2; sleep latency: 0.24 (C1), 0.21 (C2); N1: 0.29 (C1), 0.23 (C2); N2: 0.01 (C1), 0.18 (C2); number of spindles: 0.08 (C1), 0.18 (C2)) and not within the range of the large effect sizes reported for vestibular stimulation during an afternoon nap^14^. Therefore, the presence of small and medium effects cannot be ruled out with our sample size.

The lack of sleep promoting effect might be explained by sleep regulation. Bayer *et al*.^14^ applied rocking movements during an afternoon nap whereas our study investigated vestibular stimulation during nocturnal sleep. Nap sleep exhibits often a reduced sleep efficiency compared to nocturnal sleep due to reduced homeostatic sleep propensity and circadian timing^40^. With homeostatic drive and circadian phase normally permitting and promoting nocturnal sleep, sleep latency is expected to be shorter and slow wave activity (SWA) higher for nocturnal sleep compared to nap sleep. Indeed, sleep latency (first occurrence of N2) was longer and sleep efficiency lower in the nap study of Bayer *et al*.^14^ (sleep latency: 17.6 min; sleep efficiency: 73.1% in baseline nap) in comparison to nocturnal sleep in our study in a comparable population (sleep latency: 8.5 min; sleep efficiency: 96.8% in baseline night).

Therefore, in contrast to nap sleep, nocturnal sleep in good sleepers might not benefit additionally from the potential facilitating effects of vestibular stimulation. Furthermore, as SWA is dependent on the duration of prior wakefulness, SWA in nocturnal sleep might be already too high to be influenced by vestibular stimulation (saturation effect), whereas during an afternoon nap with lower sleep pressure there might be a greater potential for slow wave enhancement. This interpretation is also supported by the findings of Krystal *et al*.^41^ using electrical stimulation of the vestibular system in a sleep phase advance model of transient insomnia. Vestibular stimulation facilitated sleep onset only in a subgroup of subjects with prolonged sleep latencies during a multiple sleep latency test (≥14 min) but not in subjects with short sleep latencies. The authors concluded that the subgroup of individuals with short sleep latencies were able to fall asleep quickly regardless of circadian phase, which outweighed the effects of vestibular stimulation^41^. However, the homogeneity of our subject population did not allow for a subdivision into short and prolonged sleep latencies as our subjects exhibited rather similar latencies.

In line with the results of Bayer *et al*.^14^ a significant increase in the amount of N2 sleep and number of spindles was found for the first 2 hours of the C2 condition. However, this increase occurred only in the C2 condition during which vestibular stimulation was present for the entire duration of the analysed time window. When comparing amount of N2 sleep and number of spindles for the first 3 hours after lights out, to include a stimulation free time window, the increase was not observable any longer. Furthermore, in contrast to the findings of Bayer *et al*.^14^, the increase in number of spindles was not accompanied by a rise in spindle density. Therefore, the increase in number of spindles is likely a result of prolonged N2 sleep. A possible explanation for the increase in N2 sleep might be related to sensory processing. Vestibular stimulation at the beginning of the night might represent additional stimuli, which are introduced to the vulnerable progression of sleep and could prolong N2 sleep. In addition, as it is suggested that spindles might have sleep protecting functions due to their role in sensory input gating^42–44^, spindles could be increased to ensure maintenance of sleep during vestibular stimulation. However, these changes appear only to be temporary and not lasting beyond the stimulation period. Furthermore, as neither SWS or REM latency nor time spent in SWS or REM sleep differed among the conditions, sleep quality appears not to be negatively affected by the increase in N2 sleep. Furthermore, conditions did not differ in the total number of excluded epochs or number of sleep stage changes. This indicates that vestibular stimulation had no impact on sleep continuity or sleep quality neither while it was applied (comparison between condition B and C2) nor after it was stopped (comparison between condition B and C1).

Although in previous studies stimulation parameters were set by the experimenter conducting the study by means of applicability and comfort for the subjects, it cannot be ruled out that our approach could have influenced our findings, i.e. not all movements might have been equally effective. However, due to the small sample size a comparison of the different movement parameters was not feasible. In the studies of Woodward *et al*.^15^ and Bayer *et al*.^14^ only lateral rocking movements were applied, albeit with different amplitudes and frequencies. In our study participants were able to select between different movement axes and also choose slower stimulation frequencies. Thus, stimulation parameters are not comparable between the studies. However, in our approach individual preferences were taken into account. Furthermore, in contrast to Woodward *et al*.^15^ we did not stimulate for consecutive nights. As sleep onset was only facilitated for the second night of stimulation in the study of Woodward *et al*.^15^ stimulating for consecutive nights might need to be considered when investigating nocturnal sleep in future studies.

### Memory Performance

In line with results of previous studies^32,45–47^ overnight performance in declarative memory improved after sleep. However, overnight memory improvement exhibited similar gains in all three conditions, leading to the conclusion that vestibular stimulation did not have additional beneficial effects on memory. These findings are not surprising considering that the applied vestibular stimulation failed to enhance sleep spindles and SO over the course of the night.

The positive correlations found for spindle density, spindle number and immediate recall and delayed recall are in agreement with previous findings^17,32,48^. However, although evidence exists pointing towards a link between SO, SWA and declarative memory performance^45,49–51^, no association between these variables were observed in our study. A causal relationship between SWA and memory performance has not yet been established and memory improvement over night is dependent on various factors. Learning task characteristics, subject population or motivational factors have been shown to influence the relationship between sleep and memory improvement^52,53^. In line with this, it cannot be excluded that the greater absolute number of recalled word-pairs at delayed recall (compared to immediate recall) in our applied memory task could be related to the received feedback after immediate recall allowing for further encoding^54^. Hence, overnight improvement does not necessarily reflect a sleep related gain in memory performance but could be influenced by the received feedback. Therefore, a possible relationship between SWA and memory performance might be more complex and needs further investigation.

While our stimulation did not lead to an enhancement in sleep spindles and SO it cannot be ruled out that vestibular stimulation applied under different circumstances or in a different group of participants could positively influence memory performance if sleep quality and oscillations would be modulated.

## Conclusion and Outlook

Subjects preferred nights with motion and selected mostly slow rotational movements along the roll- or pitch-axis. Nevertheless, sleep onset was not facilitated by vestibular stimulation and no changes in spectral power and SO were observed. Overnight memory performance was enhanced, however, sleep-dependant improvement in memory performance was observed in all conditions. Nonetheless, a potential memory enhancing effect of vestibular stimulation might be induced when sleep quality and/or oscillations can be modulated with such an intervention.

Sleep regulation might be an explanation for the lack of sleep promoting effect, as nocturnal sleep in very good sleepers was investigated. Sleep under these conditions already exhibits high sleep quality (short sleep latency, high sleep efficiency), which hardly can be improved by such a stimulation. Future studies have to investigate whether vestibular stimulation might have a facilitating effect on sleep onset in a population exhibiting disturbed sleep, e.g. prolonged sleep latencies, low sleep efficiency, or reduced homeostatic sleep drive. Nevertheless, as changes in sleep only appear to be observable during stimulation, all night stimulation should be considered in future studies as well as stimulation during consecutive nights.
